# Maximizing community participation and engagement: lessons learned over 2 decades of field trials in rural Ghana

**DOI:** 10.1186/s12982-021-00110-7

**Published:** 2021-12-24

**Authors:** Sam Newton, Guus Ten Asbroek, Zelee Hill, Charlotte Tawiah Agyemang, Seyi Soremekun, Seeba Amenga Etego, Betty Kirkwood

**Affiliations:** 1 School of Public Health, Kwame Nkrumah University of Science and Technology, Private Mail Bag Kumasi, Ghana; 2 Department of Global Health, Amsterdam University Medical Centers, Location AMC, 1105 AZ Amsterdam, The Netherlands; 3 Amsterdam Institute for Global Health and Development, 1105 BP Amsterdam, The Netherlands; 4 Institute of Child Health, Amsterdam, The Netherlands; 5 Kintampo Health Research Centre, Kintampo, Ghana; 6 Department of Infectious and Tropical Diseases, London School of Hygiene and Tropical Medicine, London, UK; 7 Department of Epidemiology and Population Health, London School of Hygiene and Tropical Medicine, London, UK

**Keywords:** Community, Participation, Engagement, Research, Health, Ghana

## Abstract

**Background:**

Successful implementation of community-based research is dominantly influenced by participation and engagement from the local community without which community members will not want to participate in research and important knowledge and potential health benefits will be missed. Therefore, maximising community participation and engagement is key for the effective conduct of community-based research. In this paper, we present lessons learnt over two decades of conducting research in 7 rural districts in the Brong Ahafo region of Ghana with an estimated population of around 600,000. The trials which were mainly in the area of Maternal, Neonatal and Child Health were conducted by the Kintampo Health Research Centre (KHRC) in collaboration with the London School of Hygiene and Tropical Medicine (LSHTM).

**Methods:**

The four core strategies which were used were formative research methods, the formation of the Information, Education and Communication (IEC) team to serve as the main link between the research team and the community, recruitment of field workers from the communities within which they lived, and close collaboration with national and regional stakeholders.

**Results:**

These measures allowed trust to be built between the community members and the research team and ensured that potential misconceptions which came up in the communities were promptly dealt with through the IEC team. The decision to place field workers in the communities from which they came and their knowledge of the local language created trust between the research team and the community. The close working relationship between the District health authorities and the Kintampo Health Research Centre supported the acceptance of the research in the communities as the District Health Authorities were respected and trusted.

**Conclusion:**

The successes achieved during the past 2 decades of collaboration between LSHTM and KHRC in conducting community-based field trials were based on involving the community in research projects. Community participation and engagement helped not only to identify the pertinent issues, but also enabled the communities and research team to contribute towards efforts to address challenges.

## Background

Community based research gives us that important added value by testing scientific ideas in the real world before these ideas can be taken into policy and standard practice. Successful implementation of community based research is dominantly influenced by the participation and engagement of the local community [19]. This is both an ethical responsibility, and essential for high quality scientific outcomes [10]. A lack of participation and engagement can impact the viability of a trial, as community members may not want to participate or may place demands on the trial team that cannot be met. In some situations, this can lead to trials being stopped, this was the case in the initial trials of Pre-Exposure Prophylaxis for HIV, that were abandoned largely because a lack of communication led to rumours, suspicion and speculation [21]. Without community participation and engagement important knowledge gain and potential health benefits can be missed. Community participation and engagement also allows researchers to gain from the unique experience and knowledge of the community, who can meaningfully contribute to and inform the research process [10].

Although the importance of community participation and engagement in community trials in increasingly recognized, there is no agreement on what it actually is, or how it should be done, and there are a broad range of approaches [12, 22]. Following over two decades of conducting field trials in rural central Ghana, we wish to describe and share experiences of what we found most helpful within that context. We aim to add to the limited but growing information on community participation and engagement and also share practical examples from working with diverse and dispersed populations in rural central Ghana. The examples are taken from trials, conducted in seven districts of the Brong Ahafo region. The region has a total area of 12,274sq km with an estimated population in the research area of around 600,000, who live in rural villages and hamlets or in three big towns. The trials had an emphasis on maternal, neonatal and child health interventions and were conducted in a collaboration between the London School of Hygiene and Tropical Medicine (LSHTM) in the UK and the Kintampo Health Research Centre (KHRC) in Ghana. The context within which these trials were conducted is described in detail elsewhere [11], but for this paper it is particularly relevant to understand that we worked in a population characterized by:

- Speaking three main languages and a number of dialects,
- Different religious backgrounds: Christians, Muslims and those with traditional beliefs,
- Economic dependency on farming and small-scale businesses.

One of the strengths of this paper is that it not only focuses on community participation and engagement within a biomedical trial setting but it adds to the sparse data on what community engagement looks like in the context of trials that have been successfully implemented.. We aimed to ensure two-way communication between the researchers and the community, so that community members understood the research fully, and so that the researchers understood and addressed the communities’ concerns and needs. The original engagement methodologies evolved in KHRC as a result of diverse needs of different projects and dissemination of project outcomes. The description of the engagement strategies, what we found most helpful and the examples we present were identified through a workshop with staff involved in the trials and reviews of project documents. While there may be other things that implicitly enhanced community engagement, we identified four key explicit strategies:

1. Use formative research to understand the communities in which we work and learn from them
2. Establish a coordinating team, the information education and communication team, to conduct a series of community engagement activities with a variety of stakeholders and throughout the life of the trial,
3. Recruit fieldworkers who are from the trial communities
4. Engage with national and regional stakeholders as partners

## Using formative research and learning from the community

Most of the trials that we conducted had a formative research phase prior to implementation. Formative research provided a detailed understanding of the communities and their beliefs and practices, collected information on the communities’ perceptions of the upcoming trials, identified potential implementation problems and solutions, and potential communication channels. Data were collected from a range of respondents including regional and district stakeholders, health workers, traditional healers, grandmothers, mothers and husbands. The extent of the formative research was dependent on how much the trial involved community level behaviour change. The formative research informed the design of interventions and data collection, and was the first step in building a trusting relationship with stakeholders and communities. Details of the formative research process and outcomes are described elsewhere [1, 7, 8]. Formative research was an important step in developing the participation and engagement strategies described below.

In addition to the formal formative research, the trial teams used their knowledge of the participants when making decisions about the trial. For example the research team took the farming lifestyle of the participants into consideration in their work schedules, collecting data very early in the morning, when farming families were already up, but at a time that was long before the formal work day of KHRC had started. Another example is related to identification of compounds. In order to identify compounds, we put numbers on all houses as there were no existing house numbers on most structures. We initially thought participants may not like us writing numbers on their houses, but by listening to the community members we found out that these would be valued because it made houses easier to identify in the community as well. The only exception were numbers that had undesirable religious connotations, in one instance a house was assigned the number 666 but was given a new number because the inhabitants were not happy with that number.

## The Information, Education and Communication team

The Information, Education and Communication (IEC) team developed strategies for communication about the work of the health research centre in general—and the specific trials in particular. This team was made up of Senior Social Scientists, who had conducted the formative research for the trials, and who served as the main link between the research team and the community. Through their formative work, the team had extensive knowledge of the trial participants and could both anticipate and solve problems with the participants’ view point in mind. When it was needed the team conducted additional qualitative trials to understand communication issues. For example, it became clear that in the ObaapaVitA Trial participants had varied understanding of issues such as placebo, the team thus conducted additional research and designed strategies to address the issue [9].

Importantly the IEC team formed an integral part of the trial management team that met weekly to discuss trial progress and make strategic decisions, this meant they could provide a community perspectives to discussions, and could identify issues that they may needed to consult with, or inform the community about, at an early stage.

The IEC team developed messages and communication strategies around core issues such as introducing trials to the communities, gaining informed consent, and providing feedback to the community at the end of the trial. Study closure was an important issue we considered especially in trials where community members at received an intervention for some time. In order to reach the maximum number of people they used a multi-pronged strategy including community meetings, posters, frequently asked question booklets, visits to schools, work associations, women’s groups, announcements in churches, mosques, health facilities and radio discussion programs. The communication methods were depended on the target audience, for example in urban areas people tended not to come to community meetings so radio broadcasts were the focus, and in areas without radio coverage vans with loudspeakers were used to play messages. The team also considered the credibility of the communication channel, for example if there was a radio discussion program on the trial, a trusted health worker would usually be asked to join. The IEC team also conducted sensitization activities with groups who community members seek advice from such as traditional healers, traditional birth attendants (TBAs) and health workers.

We considered developing good trusting relationships with the community from the earliest stages of the trial as essential, and considerable time and resources were put into strategies to introduce the trials. We adopted a cascade approach first meeting with the regional and then the district political and traditional administrations, then the community level administrations, the community itself, the family and finally the individuals being asked to participate. The community meetings started with religious and political leaders, as their permission was needed to work in the communities. Local traditions such as presenting a bottle of Schnapps (alcoholic drink) to the chiefs were always followed. The alcoholic drinks were then used by the chiefs to offer prayers to God for a successful implementation of the trial. Once the leaders had been informed about the trial, they facilitated organizing a community meeting at a time most convenient for the community members which was called by the traditional local announcer, the *gong gong* beater. In the meetings issues such as the rationale for conducting the research, why their communities had been chosen for the trials and the randomization process were explained. Care was taken to use local languages and to describe technical issues such as randomization and placebo in a meaningful way. Senior research staff were always present at these meetings to provide credibility and to ensure all questions were answered correctly.

Once community meetings had been held, fieldworkers assigned to each community visited each house in turn explaining the trial first to the family and then to the individual woman who was being asked to participate. At all events community members were encouraged to ask questions, and they usually did. Given that community meetings were not attended by all community members, and that some people may feel intimidated asking questions is such a forum, special attention was paid to how fieldworkers communicated at the family level. Fieldworkers were given information sheets that explained the trial in simple language and were trained in communication skills such as active listening by the IEC team. We believe that this communication training not only improved how the fieldworkers communicated, but also highlighted to them the importance of what the community has to say.

Throughout the trials the IEC team continued to conduct routine activities such as holding focus groups to understand community perspectives, and responding to any concerns raised by the community members. An example of this were issues related to collecting blood samples in a vitamin A supplementation trial [17]. The recruitment of subjects into the trial was threatened by a rumour circulating in the community that the samples of blood which was collected for retinol assessment was intended for sale abroad for transfusion to the aged. This problem was rapidly identified and acted on by the IEC team who organised a series of community meetings to address concerns and to allay their fears about the true intention of the researchers. The IEC team explained why it was necessary for the samples to be collected using the analogy of blood sample analyses at hospitals when patients report for laboratory investigations. Some research participants were invited to visit the KHRC laboratories to observe the processing of blood samples being used for research purposes.

## Recruitment of field workers from the communities within which they lived

Field workers were recruited from the trial area and were then matched to a community based on their knowledge of the community and the language spoken. They were introduced to the community during the community meetings and then lived in the community they collected data from, and served as a link between the research centre and the community. The use of resident fieldworkers helped build rapport and trust between the community members and their fieldworker and with the trial in general. The fieldworkers became a known face in the community, were highly accessible and approachable if participants had any problems, and were involved in informal engagement activities such as attending weddings and funerals. They were given project T-shirts and bags to highlight their affiliation with the trial, and were frequently supervised in the community which helped the community see them as a trained, skilled and credible workers.

Once the trials were up and running the resident fieldworkers were one of the key communication channels for the IEC team and provided a continuous forum for communication between the community and the trial team. At monthly fieldworker meetings the IEC team provided messages for fieldworkers to give to the families they visited. Fieldworkers reported community concerns and issues to the IEC team, who then drew up a plan to address them.

There are issues with using embedded fieldworkers in the community that we needed to consider [15], first if their selection is thought to be inequitable community tensions may arise and trust in the fieldworkers may diminish. We used an open and transparent method of selecting fieldworkers where anyone could apply and with selection based on a short test. Second if the communities know the fieldworkers and their backgrounds they may question how knowledgeable they are, we tried to distinguish the fieldworkers from ordinary community members by using trial branding and community level supervision to enhance their credibility.

These fieldworkers were both men and women, there was some culturally sensitivity around men talking to women, but after discussion with the community we found that this could be overcome if interviews were conducted in the open. Field data collectors were instructed to carry out interviews in the open and not to enter the mud huts or bedrooms of the women. They visited each household once a month and where there was a need for more frequent visits they did so. We also made sure that the field workers were branded and identified with the research team and that also reduced suspicion.

## Engage with national and regional stakeholders as partners

KHRC built and maintains a strong collaboration with the health authorities at national, regional and district level. At national level key stakeholders were members of Trial Advisory Groups, and through this had significant influence on the conduct of the trials. In several trials key national, regional and district stakeholders participated in trial design workshops, where the results of the formative research were presented and key decisions made about intervention and trial design. At district level KHRC, which is under the Research and Development Division of the Ghana Health Service, worked closely within the existing health systems to carry out the research. This close working relationship between the District health authorities and the Kintampo Health Research Centre supported the acceptance of the research in the communities as the District Health Authorities were respected and trusted. The relationship with the existing health system was both formal, for example for some trials KHRC staff were seconded to the district health offices, and informal, with personal trusting relationships developing over time. We consulted on a range of stakeholders involved in the community engagement activities and held several information-gathering workshops and through an iterative process agreed what we felt were the key lessons learned and presented here.

## Dissemination

Community participation and engagement strategies and skills can build trust and reduce historical mistrust between researchers, communities, and populations being studied. It can contribute to improving research methods and assist with dissemination of findings [2]. In our case, dissemination was done at the village or district level after a project ending. There were cases however, where we had to do so at the individual level by going from house to house for various reasons such as informing women that there was no longer the need for them to continue taking supplements as the trial had ended. This was because sharing this information at the village level in some cases was deemed inadequate. In summary we modified our dissemination strategy and used the house to house approach as an opportunity to reengage with the community and increase coverage especially for new studies which were introducing a new intervention.

## Challenges

These community engagement activities took considerable organisation and resources including forming specific teams for formative research and IEC, holding community meetings, regular FGDs and training all fieldworkers on communication skills. Given the size of the study area and the multiple language groups this was complex to organise, but was deemed necessary to ensure our losses to follow up were minimised. Throughout the trials new community issues were identified that requited engagement and this was not a one time activity but a long term commitment to engage with and listen to the community and study participants.

## Conclusions

The successes achieved during the past 2 decades of the collaboration between LSHTM and KHRC in conducting community based field trials could not have been achieved without community support and participation. By listening to the community through the use of formative research, a dedicated Information, Education and Communication team, and approachable fieldworkers trained in communication skills, we were able to understand the needs of the community and identify concerns they may have about the trials. We engaged with a wide range of community members and stakeholders to ensure a wide range of views were heard. We frequently used community meetings as an engagement strategy, such meetings may alienate some community members [10], and we made sure that we also used other strategies to engage those who may not want to attend or speak at such meetings.

Over two decades of community engagement we have used a wide variety of methods and targeted a wide range of stakeholders such as community leaders, chiefs and opinion leaders. The selection of methods and stakeholders was both driven by formative research, learning from the experience and through the creative thought processes of the trial teams. Having some measure of effectiveness would allow researchers to streamline their community engagement activities, and a more empirical approach to community engagement has been called for to move away from community engagement driven by intuition, experience and opinion [16].

Two way communication between the community and the researchers was facilitated by a clear communication chain from community level to the trial management team, and a specific group, the IEC team, tasked with developing issue management plans. Engaging communities early is well recognized as a key part of community participation and engagement and we adopted a cascade approach working from the highest level stakeholders and opinion leaders down to the family. The cascade approach has been successfully used in other sites, and meeting with traditional and political leaders to gain their approval is important where community consent is needed before individuals can make decisions, and because community approval provides a sense of security and the building blocks for a trusting relationship with the trial team [5].

At the individual level having resident fieldworkers who were available and approachable allowed mutual understanding between the community and the trial team to develop, as the field workers were considered part of the community.

KHRC has a long term presence in the trial area and our formative research shows that the population generally has a high level of trust of KHRC as an institution. High levels of trust have also been found in other areas with high levels of exposure to research [13, 14]. This overarching trust influences how communities respondent to, and internalize, the information provided through community engagement strategies [3]. Where trust is high communities may not feel they need to process or question information provided by the trial, and may become passive rather than active participants.

A lot of the success achieved by the research team can also be attributed to working closely with the District health authorities and community members to ensure acceptance of the research in the communities. Mechanisms that ensure systematic involvement of legitimate representatives of the affected community as partners in research are the only way to ensure that future trials will proceed in a more productive way [4].

There is the need for transparency about all of the project’s activities, engagements and experiences. This involves informing the public of challenges and potential risks in the project [6]. Even though the community involvement process is long, laborious and ever-evolving, effective community participation and engagement requires institutional leadership support, adequate funding and commitment by researchers [20]. When this is well done, community engagement processes affords significant opportunities for improving maternal and child health and for facilitating change in the community community [23]. Community participation and engagement helps not only to identify the pertinent issues, but also enables the communities concerned to contribute towards efforts to address the challenges. Such a participatory approach goes a long way in promoting mutual trust between researchers and communities and creates a sense of ownership of the research [18].

Much of what is described in this paper is specific to the context in which we were working, both in terms of community structure and the nature of the trials themselves Community participation and engagement needs to vary by context, but by sharing some of our experience we hope to provide other researchers with food for thought as they develop their own plans.

## Abbreviations

IEC Information: Education and Communication
KHRC: Kintampo Health Research Centre
LSHTM: London School of Hygiene and Tropical Medicine
TBA: Traditional Birth Attendants
